# The effect of evening swimming exercise on sleep parameters in male swimmers

**DOI:** 10.14814/phy2.71050

**Published:** 2026-08-12

**Authors:** Hiroki Furukawa, Tomotaka Natsui, Takenobu Chugun, Chihiro Tomiishi, Rin Mizuno, Yuri Maeoka, Kazushige Goto

**Affiliations:** ^1^ Graduate School of Sports and Health Science Ritsumeikan University Kusatsu Japan; ^2^ Department of Health and Physical Education, Faculty of Health and Medical Technology Kawasaki University of Medical Welfare Kurashiki Japan

**Keywords:** body temperature, sleep, swimming, vagal nerve, vigorous exercise

## Abstract

We evaluated the effects of evening swimming exercise on sleep variables in male swimmers. In a crossover design, 12 competitive male swimmers completed two trials (6:30–8:30 pm): the high‐intensity swimming exercise condition (EX) and no‐exercise condition (CON). Sleep quality was assessed using electroencephalography, and core body temperature (CBT) was evaluated by the pill type thermometer. Average CBT during the intervention period was significantly higher in EX (37.8°C ± 0.3°C) compared with CON (37.1°C ± 0.2°C, *p <* 0.05). However, at bedtime (11:00 pm), CBT did not differ significantly between the conditions (37.2°C ± 0.2°C in EX vs. 37.2°C ± 0.3°C in CON, *p >* 0.05). In EX, the duration and the percentage of rapid eye movement (REM) sleep were significantly lower (81.4 ± 26.7 min in EX vs. 107.6 ± 39.5 min in CON, *p <* 0.05), and wake after sleep onset was significantly higher (23.5 ± 39.6 min in EX vs. 3.4 ± 2.5 min in CON, *p <* 0.05). However, other sleep variables did not differ significantly between the conditions (*p >* 0.05). These findings suggest that evening swimming exercise induced selective rather than generalized impairments in sleep.

## 1 INTRODUCTION

Sleep is an indispensable physiological behavior in humans and plays essential roles in memory consolidation and physiological recovery (Diekelmann & Born, 2010; Fallon, 2007; King et al., 2017; Venter, 2014). Athletes who perform high‐intensity and prolonged training on a daily basis particularly require adequate sleep to facilitate recovery from daily exercise (Bird, 2013; Laursen, 2010). Although several strategies for promoting post‐exercise recovery have been proposed, sleep is considered the most important recovery modality following exercise (Charest & Grandner, 2020; Doherty et al., 2021; Fallon, 2007). Previous studies have demonstrated that insufficient sleep duration impairs physical performance and recovery on the following day (Oliver et al., 2009; Rae et al., 2017). However, increasing sleep duration is often challenging for athletes because of demanding and time‐restricted daily schedules. Therefore, optimizing sleep quality is essential for enhancing athletic performance and maintaining overall health.

Some athletes have to perform daily exercise during the evening or at night. This situation is particularly common among student athletes and working athletes who attend classes or work during the daytime. However, high‐intensity exercise performed in the evening impairs sleep quality (Aloulou et al., 2020; Juliff et al., 2018; Oda & Shirakawa, 2014; Roberts et al., 2019; Wong et al., 2013). Oda and Shirakawa (2014) demonstrated that 40 min of high‐intensity running exercise performed at 9:20 pm to 10:00 pm with 80% of maximal heart rate (HR) significantly increased sleep onset latency (SOL) and decreased both total sleep time (TST) and sleep efficiency (SE). Similarly, Aloulou et al. (2020) reported that vigorous running designed to mimic trail running, performed at 9:00 pm, increased the proportion of light sleep stages (N1 + N2). The decline in sleep quality observed in these studies may have been attributed to elevated core body temperature (CBT) and increased sympathetic nervous system activity accompanied by an elevated HR (Frimpong et al., 2021; Uchida et al., 2012).

CBT or HR is known to be affected by the exercise environment. During exercise in water, venous return is augmented due to hydrostatic pressure (Wilcock et al., 2006). Consequently, the exercise‐induced increase in HR is smaller during water‐based exercise than during land‐based exercise (DiCarlo et al., 1991). Furthermore, heat conductivity is greater during water‐based exercise than during land‐based exercise (Jimenez‐Perez et al., 2021; Nielsen & Davies, 1976), thereby attenuating the increase in CBT (Craig Jr & Dvorak, 1969). Additionally, a previous study showed that water immersion after a land‐based exercise enhanced reactivation of parasympathetic nervous activity (Al Haddad et al., 2010). These physiological responses are inconsistent with the mechanisms typically associated with sleep disturbance. Therefore, water‐based exercise may help attenuate excessive increases in CBT and HR. These alterations may promote recovery of autonomic nervous activity, thereby reducing sleep disturbance.

Overall, evening high‐intensity exercise under aquatic conditions may not negatively affect subsequent sleep. However, whether evening high‐intensity swimming exercise affects sleep quality remains unclear. Therefore, we determined the effects of evening high‐intensity exercise in water on sleep quality in college swimmers. We hypothesized that evening swimming exercise would not reduce sleep quality.

## 2 MATERIALS AND METHODS

### 2.1 Participants

In total, 12 well‐trained competitive male college swimmers who performed regular swimming training 6 days per week were recruited. The sample size was calculated using G*power (version 3.1.9.7), indicating that 12 participants were required (effect size = 0.90, α = 0.05, power [1‐β] = 0.8). SE was selected as the primary outcome variable. The effect size was estimated based on previous studies comparing SE following evening high‐intensity exercise with resting/control condition (e.g., Flausino et al., 2012; Oda & Shirakawa, 2014; Vlahoyiannis et al., 2021). When multiple exercise conditions were included, the high‐intensity exercise condition was selected and compared with the resting/control condition. Consequently, the mean calculated effect size was 0.87, and an effect size of 0.90 was adopted for the power analysis.

Participants were informed of the experimental procedures, the purpose of the study, and the potential risks associated with participation. Written informed consent was obtained from all participants before enrollment. The exclusion criteria were: diagnosed sleep disorders (sleep apnea, insomnia, or narcolepsy); regular use of sleep medications; presence of cardiovascular disease, diabetes, hypertension, or depression; travel across time zones within 1 month before the start of the study; and shift work or smoking.

### 2.2 Experimental overview

Our study was conducted using a randomized crossover design. The experimental protocol consisted of a familiarization session followed by two experimental conditions performed on separate days: a high‐intensity swimming practice condition (EX) and a no‐exercise control condition (CON). The two conditions were separated by ≥1 week. The measurements in each condition were scheduled on the same day of the week (weekdays) during the pre‐season period.

**FIGURE 1:**
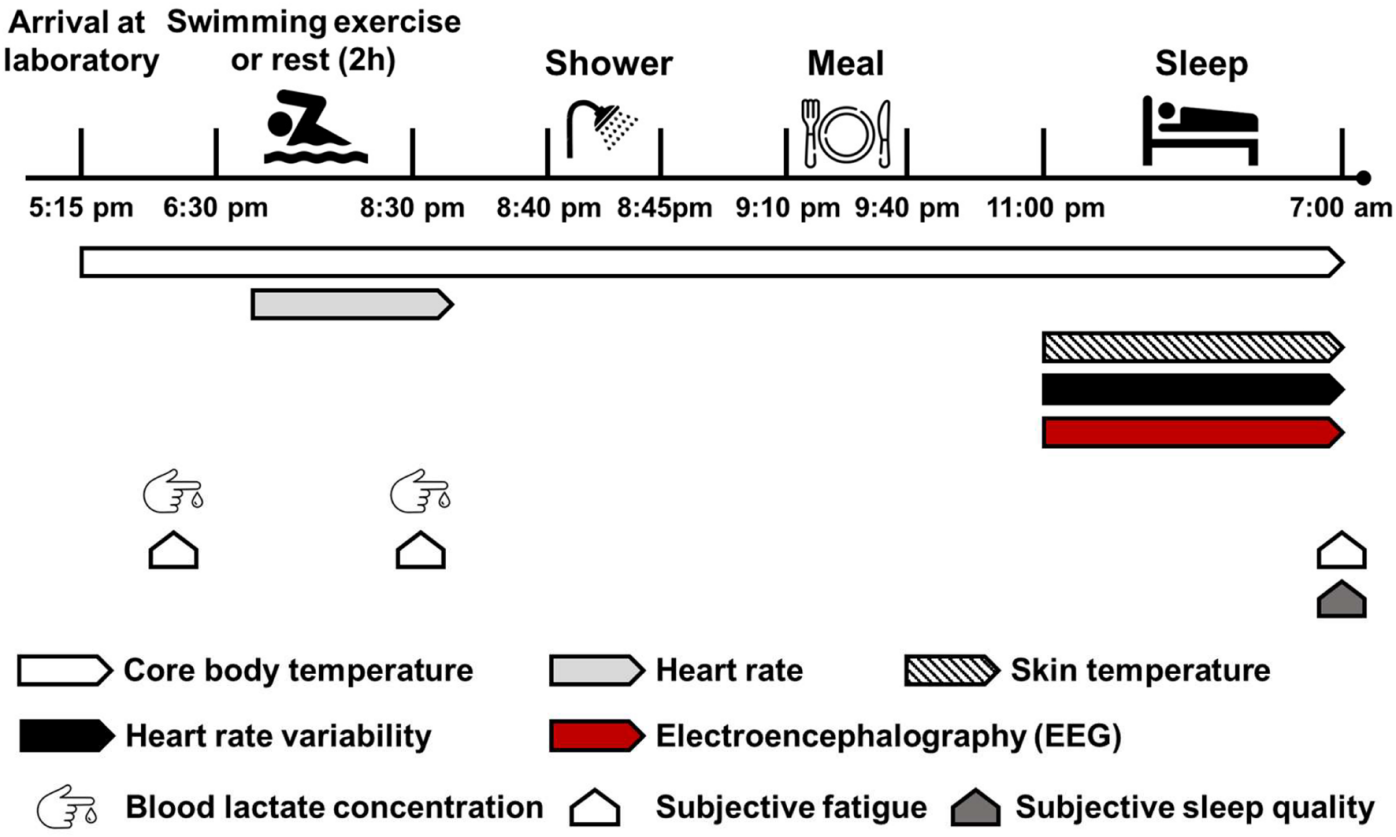
Experimental overview and timing of measurements.

In both conditions, participants were instructed to avoid any training other than the prescribed exercise session throughout the intervention day. On the testing day for EX or CON, participants arrived at the laboratory at 5:15 pm, and baseline measurements were obtained, including blood lactate concentration and subjective fatigue assessed using a visual analog scale (VAS). Subsequently, participants completed 2 h of the assigned intervention protocol (2 h of swimming practice in EX or an equivalent period of rest in CON). After the intervention, participants showered for 5 min and then consumed a prescribed dinner (1173 kcal; protein, 38.4 g; fat, 35 g; carbohydrate, 179 g) at 9:10 pm. Following dinner, participants moved to accommodation located on the university campus (Figure 1).

#### 2.2.1 Familiarization session

The familiarization session was conducted at least 1 week before the first experimental condition (CON or EX). This session was performed to familiarize participants with sleeping in the accommodation room located on the university campus. The room temperature was set at 23°C and the room was shielded from external light by curtains, without putting devices such as televisions or monitors. During sleep, participants wore a portable electroencephalography (EEG) device and sensors for monitoring HR variability (HRV) and skin temperature (Tsk). In addition, body composition variables, including height, weight, and body mass index, were assessed. Subjective sleep quality was evaluated using the Pittsburgh Sleep Quality Index (Buysse et al., 1989), and chronotype was assessed using the Morningness–Eveningness Questionnaire (Horne & Ostberg, 1976).

#### 2.2.2 Intervention

Each intervention (swimming practice in EX and an equivalent rest period in CON) was conducted between 6:30 pm and 8:30 pm. The swimming practice protocol in EX consisted of a warm‐up session, a technique session, a session using only the upper or lower limbs, and a high‐intensity session followed by a 10 min active recovery period in the water. The high‐intensity session consisted of intermittent sprint training (10 × maximal 50‐m freestyle sprints interspersed by 4 min, measured from the onset of the preceding sprint). During the active recovery period, participants were instructed to swim slowly with sufficient strokes. The water temperature during exercise was maintained at 29°C. In CON, participants remained seated at rest in a room maintained at 25°C for 2 h while reading books or studying.

#### 2.2.3 Evaluation of sleep quality

After arrival at the accommodation, the equipment for EEG, Tsk, and HRV measurements was set up. Between 10:00 pm and 11:00 pm, participants remained seated at rest without a specific activity. The use of electronic devices was prohibited from 10:00 pm to 7:00 am. Participants spent time in bed from 11:00 pm to 7:00 am.

Sleep quality during the night was assessed using a portable two‐channel EEG device (ZA‐X, Proassist Ltd., Osaka, Japan). The device consists of four electrodes: two electrodes measure EEG signals, one measures electromyography, and one measures electrooculography. Electrodes were placed above and lateral to the outer canthus of the left eye and on the right mastoid. Two additional electrodes were placed below and slightly lateral to the outer canthus of the right eye and above the chin. Sleep stages were analyzed in 30‐s epochs. A previous study (Nonoue et al., 2017) reported that sleep variables obtained from this device showed strong correlations with those obtained using polysomnography (e.g., TST: *r* = 0.980, WASO: *r* = 0.941, *p* < 0.001) as well as high inter‐scorer concordance rate (*κ* = 0.80, *p* < 0.001), and acceptable agreement in Bland–Altman analyses. These values were comparable to those obtained using standard polysomnography (*κ* = 0.85, *p* < 0.001).

The EEG data obtained during sleep were classified into five stages: wakefulness, rapid eye movement (REM) sleep, and non‐REM sleep stages 1–3 (N1–N3). In addition, TST, SE, SOL, and wake after sleep onset (WASO) were calculated. In the present study, WASO was defined as the total duration of wakefulness occurring after sleep onset and before the final awakening. Therefore, the interval between the final awakening and the scheduled wake‐up time (7:00 am) was not included for WASO.

In the present study, the EEG data, HR and HRV variables were assessed over the whole night and during the first 90 min after bedtime, because stage N3 predominates during the early phase of nocturnal sleep and autonomic disturbances following high‐intensity exercise are expected to be most pronounced during this period (Oda & Shirakawa, 2014). Moreover, exercise‐induced physiological responses such as elevated CBT and higher HR return to baseline within 1–3 h after evening exercise (Aloulou et al., 2020).

### 2.3 Measurements

#### 2.3.1 Blood lactate concentrations

Blood lactate concentrations were measured before and after the intervention (2 h of swimming exercise in EX or 2 h of rest in CON). Blood samples were obtained from the fingertip (approximately 0.3 μL) and analyzed using a lactate sensor (Lactate Pro2; Arkray Inc., Kyoto, Japan). HR was recorded every min during the 2‐h intervention using a wireless HR monitor (Polar H10 N; Polar Electro Oy, Kempele, Finland).

#### 2.3.2 Core body temperature

At 3:00 pm, participants ingested a radio telemetric pill (e‐Celsius® Performance; BodyCap, Normandy, France). This device has been used widely in previous studies to monitor CBT during sleep and swimming exercise (Dominiak et al., 2021; Racinais et al., 2022). CBT was recorded continuously from 30 min before the intervention (6:00 pm) until the following morning (7:00 am). The recorded data were averaged at 1‐min intervals.

#### 2.3.3 Heart rate variability

To evaluate nocturnal autonomic nervous system activity, HRV was measured using an HR sensor (WHS‐1; Union Tool Co., Tokyo, Japan) from 30 min before bedtime (10:30 pm) until the following morning (7:00 am). Subsequently, HRV data were analyzed using automatic filtering software (RRI Analyzer 2; Union Tool Co., Tokyo, Japan). All data were sampled in 5 min epochs. The following HRV variables were assessed: frequency‐domain indices, including low‐frequency power (LF), high‐frequency power (HF), and the LF/HF ratio; time‐domain indices, including the SD of NN (R–R) intervals (SDNN), the root mean square of successive differences in R–R intervals (RMSSD), the number of successive R–R interval differences greater than 50 ms (NN50), and the percentage of NN50 relative to all NN intervals (pNN50); and HR. The same sensor (WHS‐1; Union Tool Co., Tokyo, Japan) has been previously utilized to evaluate HRV (Kobayashi et al., 2025; Murakami et al., 2025; Tanaka et al., 2024).

#### 2.3.4 Skin temperature

Tsk was recorded from 10:30 pm to 7:00 am using a data logger (N543; Nikkiso Therm Co., Ltd., Tokyo, Japan). The data were obtained at 0.5 Hz. Electrodes were attached to the center of the back of the right hand, the right foot, and the chest. The Tsk value measured at the subclavian chest region was defined as the proximal temperature, whereas the average temperature of the hand and foot was defined as the distal temperature. The distal‐proximal gradient (DPG) was calculated by subtracting the proximal temperature from the distal temperature (Ichiba et al., 2019).

#### 2.3.5 Subjective rating

Subjective fatigue was assessed using a 100‐mm VAS before the intervention (exercise or rest), immediately after completion of the 2‐h intervention, and after waking the following morning. Participants evaluated subjective sleep parameters, including ease of falling asleep, perceived sleep quantity, overall sleep quality, and ease of awakening, using a VAS immediately after the prescribed wake‐up time (7:00 am). In addition, subjective sleepiness was assessed using the Karolinska Sleepiness Scale in the morning after the sleep measurement (Åkerstedt & Gillberg, 1990).

### 2.4 Statistical analyses

All data are presented as mean ± SD. The assumption of normality for all variables was evaluated using the Shapiro–Wilk test. For changes in blood lactate concentration, CBT and Tsk, two‐way repeated‐measures analysis of variance was used to examine interaction effects (condition × time) and main effects (condition and time). When a significant interaction or main effect was observed, Tukey's post hoc test was performed to identify specific differences. Comparisons of HR, HRV, and sleep, the rate of CBT decline including the entire night sleep and first 90 min, variables between conditions were performed using a paired *t*‐test for normally distributed data or the Wilcoxon signed‐rank test for non‐normally distributed data. Furthermore, correlations were calculated using Pearson's correlation analysis between CBT decline and HRV parameters. A two‐sided *p*‐value <0.05 was considered statistically significant. All statistical analyses were performed using SPSS (ver 29; IBM Corp., Armonk, NY, USA).

## 3 RESULTS

### 3.1 Characteristics of participants

Table 1 shows the characteristics of the participants in the present study.

**TABLE 1:** Characteristics of participants.

Age (years)	19.7 ± 1.4
Height (cm)	174.7 ± 4.7
Weight (kg)	68.6 ± 5.0
BMI	22.5 ± 1.9
PSQI	4.8 ± 1.4
Morning‐evening questionnaire	49.3 ± 11.5
World Aquatic Point (points)	721.0 ± 65.0

*Note*: Values are mean ± SD.

Abbreviations: BMI, body mass index; PSQI, Pittsburgh Sleep Quality Index.

### 3.2 Blood lactate concentration and HR during exercise and rest period

Significant interaction (condition × time) and main effects of condition and time were found for blood lactate concentration (*p <* 0.01, respectively). Blood lactate concentration before intervention did not differ significantly between conditions (CON: 2.4 ± 1.5 mmol/L; EX: 1.9 ± 0.6 mmol/L, *p* > 0.05). However, blood lactate concentrations immediately after swimming exercise or rest were significantly higher in EX (20.5 ± 3.4 mmol/L) compared to CON (1.8 ± 0.7 mmol/L, *p <* 0.05). Maximum HR during the intervention was significantly higher in EX (172 ± 9 bpm) compared to CON (82 ± 14 bpm, *p <* 0.01).

### 3.3 Core body temperature

**FIGURE 2:**
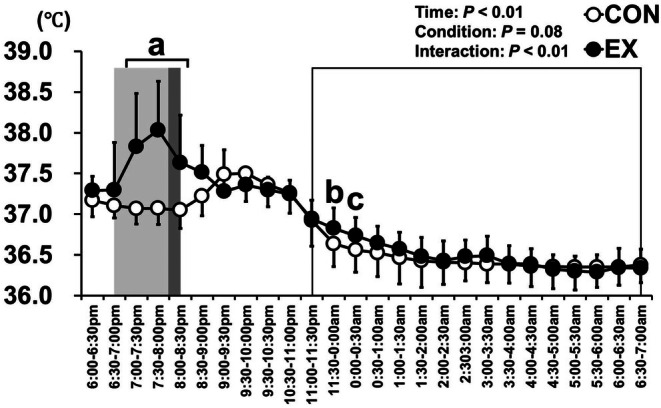
Change in CBT. Values are presented as means ± SD. a: Significant difference vs. CON (*p* < 0.01), b: significant difference vs. CON (*p* = 0.01), c: significant difference vs. CON (*p* = 0.04). The light gray bar represents during swimming exercise. The dark bar represents during active recovery in water. The open square represents during sleep.

Figure 2 shows changes in CBT. Data from one participant was excluded due to sensor error, leaving 11 participants for analysis. No significant main effect of condition was observed; however, significant interaction (condition × time) and main effects of time were detected. CBT increased significantly during swimming exercise in EX, whereas no change was observed in CON. At bedtime (11:00 pm), CBT did not differ significantly between CON (37.3 ± 0.3°C) and EX (37.2°C ± 0.2°C, *p >* 0.05). During sleep, no significant differences were observed between conditions except for the period from 11:30 pm to 0:30 am, when CBT was higher in EX than in CON (*p* < 0.05). However, the rate of CBT decline during the first 90 min after bedtime did not differ significantly between the two conditions (CON: 0.006 ± 0.004°C/min; EX: 0.005 ± 0.004°C/min, *p* > 0.05). Furthermore, no significant correlation was observed between the decline in CBT and HR (*r* = −0.163, *p* > 0.05), LF (*r* = −0.066, *p* > 0.05), HF (*r* = −0.372, *p* > 0.05), LF/HF (*r* = 0.264, *p* > 0.05), SDNN (*r* = 0.196, *p* > 0.05), RMSSD (*r* = −0.311, *p* > 0.05), NN50 (*r* = −0.154, *p* > 0.05), %NN50 (*r* = −0.149, *p* > 0.05) and total power (*r* = 0.092, *p* > 0.05) during the first 90 min of sleep, respectively.

### 3.4 Sleep quality

#### 3.4.1 Whole‐night sleep

Table 2 shows sleep variables for the whole night. TST, SE, and SOL did not differ significantly between conditions. Similarly, the duration and percentage of non–REM sleep stages (N1, N2, and N3) did not differ between conditions. However, WASO was significantly higher in EX compared to CON (*p <* 0.05). In addition, participants in EX exhibited significantly shorter REM sleep durations and lower REM sleep percentages compared to those in CON (*p <* 0.05).

**TABLE 2:** Sleep parameters during whole night sleep (11:00 pm–7:00 am).

	CON	EX	*p*‐Value
TST (min)	462.0 ± 11.9	441.2 ± 43.6	0.21
SE (%)	96.2 ± 2.5	91.8 ± 9.1	0.18
SOL (min)	12.1 ± 11.5	13.5 ± 11.5	0.81
WASO (min)	3.4 ± 2.5	23.5 ± 39.6^a^	0.01
REM sleep time (min)	107.6 ± 39.5	81.4 ± 26.7^a^	0.02
% of REM sleep (%)	23.1 ± 8.4	17.5 ± 5.6^a^	0.02
N1 sleep time (min)	7.8 ± 9.1	7.0 ± 4.8	0.50
% of N1 sleep (%)	1.7 ± 2.0	1.8 ± 4.8	0.51
N2 sleep time (min)	243.2 ± 44.7	251.0 ± 38.9	0.48
% of N2 sleep (%)	52.3 ± 9.6	54.0 ± 8.1	0.48
N3 sleep time (min)	103.5 ± 7.6	101.8 ± 12.0	0.35
% of N3 sleep (%)	22.0 ± 1.6	21.9 ± 2.9	0.93

*Note*: Values are means ± SD. Data from one participant were excluded due to sensor error, leaving 11 participants for statistical analyses.

Abbreviations: N1, non‐REM sleep stage 1; N2, non‐REM sleep stage 2; N3, non‐REM sleep stage 3; REM, rapid eye movement; SE, sleep efficiency; SOL, sleep onset latency; TST, total sleep time; WASO, wake after sleep onset.

^a^ Significant difference vs. CON (*p* < 0.05).

#### 3.4.2 First 90 min of sleep

Table 3 shows sleep variables for the first 90 min. Consistent with whole‐night findings, WASO was significantly higher in EX compared to CON (*p <* 0.05). No significant differences were observed in the duration or percentage of any sleep stage (REM or N1–N3) between conditions.

**TABLE 3:** Sleep parameters during first 90 min sleep (11:00 pm–0:30 am).

	CON	EX	*p*‐Value
WASO (min)	0.5 ± 1.2	6.0 ± 10.5^a^	0.03
REM sleep time (min)	3.2 ±4.3	2.6 ± 4.6	0.78
% of REM sleep (%)	4.1 ± 5.7	3.2 ± 5.7	0.72
N1 sleep time (min)	6.1 ± 8.6	3.5 ± 4.1	0.58
% of N1 sleep (%)	7.3 ± 9.9	4.5 ± 4.6	0.72
N2 sleep time (min)	29.9 ± 9.9	32.6 ± 10.7	0.56
% of N2 sleep (%)	38.5 ± 11.7	42.8 ± 13.1	0.42
N3 sleep time (min)	37.2 ± 11.5	30.8 ± 13.8	0.24
% of N3 sleep (%)	48.3 ± 14.2	40.3 ± 17.6	0.18

*Note*: Values are means ± SD. Data from one participant were excluded due to sensor error, leaving 11 participants for statistical analyses.

Abbreviations: N1, non‐REM sleep stage 1; N2, non‐REM sleep stage 2; N3, non‐REM sleep stage 3; REM, rapid eye movement; WASO, wake after sleep onset.

^a^ Significant difference vs. CON (*p* < 0.05).

### 3.5 Heart rate and heart rate variability

#### 3.5.1 Whole‐night sleep

Table 4 shows HR and HRV during whole‐night sleep. HR was significantly higher in EX compared with CON (*p <* 0.05). No significant differences were observed between conditions for frequency‐domain indices (total power, LF, HF, and LF/HF ratio) or time‐domain indices (SDNN, RMSSD, NN50, and pNN50).

**TABLE 4:** HR and HR variability during whole night sleep (11:00 pm–7:00 am).

	CON	EX	*p*‐Value
HR (bpm)	49 ± 5	53 ± 5^a^	0.02
Total power (ms^2^)	10,651 ± 1330	9416 ± 1242	0.36
LF (ms^2^)	2972 ± 1131	2564 ± 970	0.36
HF (ms^2^)	2495 ± 1614	1833 ± 878	0.28
LF/HF (unit)	1.4 ± 0.6	1.5 ± 0.2	0.54
SDNN (ms)	112.8 ± 23.3	105.9 ± 19.7	0.26
RMSSD (ms)	97.2 ± 31.1	85.8 ± 18.6	0.17
NN50 (number)	123.7 ± 30.0	115.7 ± 31.1	0.44
%NN50 (%)	52.1 ± 15.0	45.7 ± 13.9	0.23

*Note*: Values are means ± SD. Data from 3 participants was not collected due to sensor error; 9 participants data were finally utilized for statistical analyses.

Abbreviations: HF, high‐frequency power; HR, heart rate; LF, low‐frequency power; NN50, the number of differences in successive RR intervals greater than 50 ms; pNN50, percentage of NN50 relative to all NN intervals; RMSSD, root mean square of successive differences in RR intervals; SDNN, standard deviation of the NN (R–R) intervals.

^a^ Significant difference versus CON (*p* < 0.05).

#### 3.5.2 First 90 min of sleep

Table 5 shows HR and HRV during the first 90 min of sleep. HR and the LF/HF ratio were significantly higher in EX compared to CON (*p <* 0.05). In contrast, total power, LF, HF, SDNN, RMSSD, NN50, and pNN50 were significantly lower in EX compared to CON (*p <* 0.05).

**TABLE 5:** HR and HR variability during first 90 min sleep (11:00 pm–0:30 am).

	CON	EX	*p*‐Value
HR (bpm)	52 ± 6	63 ± 10^a^	0.01
Total power (ms^2^)	10,040 ± 8039	4710 ± 2591^a^	0.02
LF (ms^2^)	3195 ± 2998	1252 ± 599^a^	0.01
HF (ms^2^)	2637 ± 1970	1046 ± 794^a^	0.01
LF/HF (unit)	1.3 ± 0.7	1.7 ± 0.7^a^	0.02
SDNN (ms)	101.3 ± 34.1	71.7 ± 21.5^a^	0.02
RMSSD (ms)	102.4 ± 57.3	55.6 ± 21.5^a^	0.01
NN50 (number)	122.8 ± 25.8	79.6 ± 48.3^a^	<0.01
%NN50 (%)	49.1 ± 16.5	28.1 ± 19.9^a^	0.01

*Note*: Values are means ± SD. Data from 3 participants was not collected due to sensor error; 9 participants data were finally utilized for statistical analyses.

Abbreviations: HF, high‐frequency power; HR, heart rate; LF, low‐frequency power; NN50, the number of differences in successive RR intervals greater than 50 ms; pNN50, percentage of NN50 relative to all NN intervals; RMSSD, root mean square of successive differences in RR intervals; SDNN, standard deviation of the NN (R‐R) intervals.

^a^ Significant difference versus CON (*p* < 0.05).

### 3.6 Skin temperature

**FIGURE 3:**
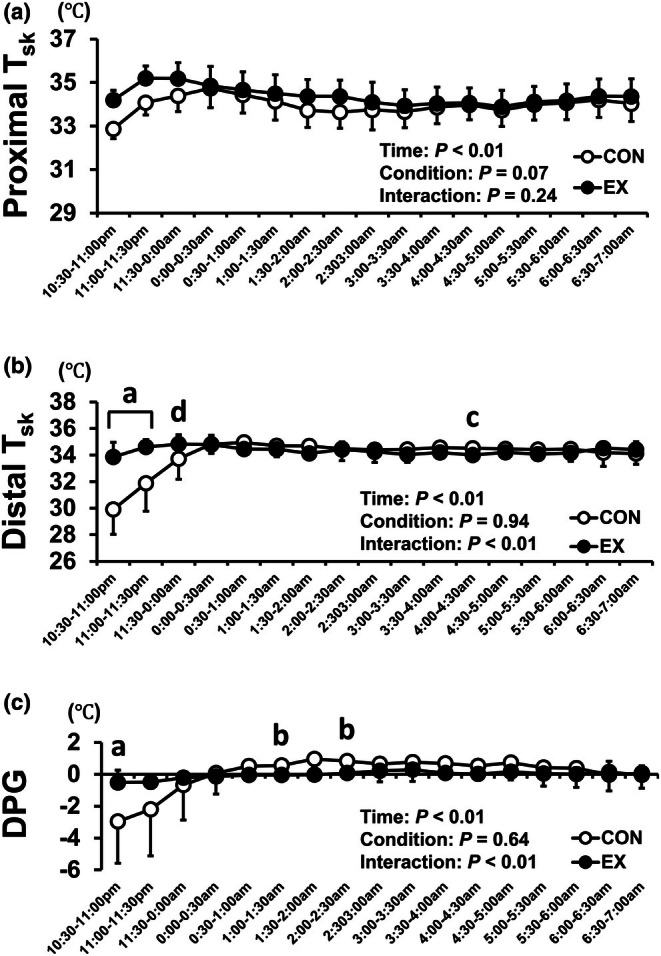
Nocturnal changes in Tsk. Proximal skin temperature during sleep (a), distal skin temperature during sleep (b), and distal—proximal gradient (DPG, c), respectively. Values are means ± SD. a: Significant difference vs. CON (*p* < 0.01), b: significant difference vs. CON (*p* = 0.02), c: significant difference vs. CON (*p* = 0.03), d: significant difference vs. CON (*p* = 0.04).

Figure 3 shows changes in Tsk values. Proximal Tsk demonstrated a significant main effect of time; however, no significant main effect of condition or interaction (condition × time) was observed. Distal Tsk and the DPG exhibited no significant main effect of condition; however, a significant interaction (condition × time) was detected. Distal Tsk was significantly higher in EX compared to CON at bedtime, 11:30 pm, and 0:00 am (*p <* 0.05), whereas it was significantly lower in EX at 4:30 am (*p <* 0.05). DPG was significantly higher in EX compared to CON at bedtime (*p <* 0.05); however, it was significantly lower in EX compared to CON at 1:30 am and 2:30 am (*p <* 0.05).

### 3.7 Subjective parameters

Perceived fatigue before and after the intervention demonstrated a significant interaction (condition × time, *p <* 0.01) and significant main effects of condition and time (*p <* 0.01). Before the intervention, no significant difference in subjective fatigue between conditions was observed (15 ± 13 mm in EX vs. 14 ± 13 mm in CON, *p* > 0.05). However, immediately after the intervention, fatigue scores were significantly higher in EX (94 ± 6 mm) compared to CON (17 ± 14 mm, *p <* 0.05). This difference remained significant the following morning (29 ± 12 mm in EX vs. 11 ± 8 mm in CON, *p <* 0.05).

Table 6 shows subjective sleep parameters. No significant differences were observed between conditions for subjective ratings of ease of falling asleep, sleep quantity, overall sleep quality, ease of awakening, or subjective sleepiness.

**TABLE 6:** Subjective sleep parameters.

	CON	EX	*p*‐Value
Ease of falling asleep (mm)	66.0 ± 24.9	55.75 ± 27.6	0.33
Sleep quantity (mm)	71.1 ± 28.3	63.8 ± 25.2	0.47
Overall sleep quality (mm)	67.3 ± 30.7	63.3 ± 27.2	0.72
Ease of awakening (mm)	71.8 ± 25.3	71.4 ± 20.6	0.96
Subjective sleepiness (unit)	4.3 ± 2.1	4.8 ± 2.0	0.51

*Note*: Values are means ± SD.

## 4 DISCUSSION

To our knowledge, this study is the first to examine the effects of evening high‐intensity swimming exercise on sleep quality in male swimmers. Our findings demonstrated that evening swimming exercise significantly increased WASO and decreased the duration of REM sleep compared with the control condition without swimming exercise, accompanied by elevated nocturnal HR, transient reductions in HRV, and a transient increase in CBT during the initial phase of sleep. However, most sleep variables, including SE, SOL, and the duration of stage N3 sleep, were not affected by evening swimming exercise. Contrary to our hypothesis, evening high‐intensity swimming exercise increased WASO and reduced REM sleep with concomitant elevated HR, indicating selective impairments in sleep quality. However, most other sleep variables, including SE, SOL, and the duration of stage N3 sleep, were preserved. These findings suggest that evening swimming exercise induced selective rather than generalized impairments in sleep.

We assessed nocturnal sleep quality using EEG following high‐intensity swimming exercise performed in the evening (6:30–8:30 pm). Our findings partially support previous studies demonstrating impaired sleep quality following intensive exercise or prolonged land‐based exercise performed in the evening or at night (Aloulou et al., 2020; Driver et al., 1994; Juliff et al., 2018; Leota et al., 2025; Oda & Shirakawa, 2014; Wong et al., 2013). Similar to previous studies, we observed elevated HR, reduced HRV during the first 90 min of sleep, increased WASO, and transiently elevated CBT during the early phase of sleep. Oda and Shirakawa (2014) reported that 40 min of high‐intensity treadmill running at night (9:20–10:00 pm) significantly increased CBT and HR at bedtime. Consequently, SOL increased significantly, whereas TST and SE decreased. A decline in CBT before bedtime facilitates sleep onset (Kräuchi, 2007; Murphy & Campbell, 1997), whereas a blunted decline in CBT at bedtime attenuates parasympathetic nervous activity during sleep (Bigalke et al., 2023; Stutz et al., 2019). In the present study, while CBT at bedtime did not differ between conditions, CBT remained significantly higher during the first hour (11:30 pm to 0:30 am) of sleep in EX. During this early phase of sleep, HR remained elevated and HRV was reduced. Although elevated CBT coincided temporally with impaired HRV, no significant correlations were observed between the decline in CBT and any HRV parameters during the first 90 min of sleep. Therefore, the higher CBT observed during the early phase of sleep does not appear to fully explain the impaired autonomic regulation observed in EX.

In our study, evening swimming exercise resulted in significantly higher HR over the night and lower HRV only in the first 90 min during sleep. However, the absolute and relative durations of stage N3 sleep were preserved. These findings suggest that evening swimming exercise in the present study affected selective changes in sleep architecture‐related and autonomic nerve activity (e.g., increased WASO and decreased REM sleep, elevated HR with decreased HRV), however, other variables (e.g., SE, SOL, time of N3) were not altered. Notably, the absence of a difference in CBT at bedtime between conditions may be attributable to rapid reduction during active recovery in water following the main strenuous swimming session. Heat dissipation through peripheral vasodilation in distal regions (hands and feet) facilitates sleep initiation (Kräuchi, 2007; Murphy & Campbell, 1997). This process increases the DPG, which promotes sleepiness (Kräuchi et al., 1999). In our study, distal Tsk and DPG values were significantly higher in EX compared to CON immediately before sleep onset (10:30–11:00 pm). Therefore, heat dissipation before bedtime appeared to be enhanced in EX, which may explain why no significant difference in CBT at bedtime was observed between the conditions. However, one possible explanation is the difference in the timing of exercise cessation before bedtime. Significant impairments in sleep quality were observed in previous studies in which exercise ended 1–2 h before bedtime (Aloulou et al., 2020; Oda & Shirakawa, 2014), while the exercise in the present study was completed 2.5 h before bedtime. A previous study revealed that, even when an exercise intensity is matched, a shorter interval between the end of the exercise and bedtime was associated with greater sleep disturbance (Leota et al., 2025). Therefore, compared to these previous studies, the participants in the present study may have had a longer recovery period to maintain sleep quality.

Although SE, SOL, and the duration of stage N3 sleep did not change significantly following swimming exercise, the participants in EX exhibited a significantly higher HR throughout the night and lower HRV during the initial 90 min of sleep. Increased exercise workload (greater cardiac strain) disrupts autonomic regulation during sleep and impairs sleep quality (Leota et al., 2025). In addition, reductions in HRV during the post‐exercise period depend on the intensity, duration, and type of exercise performed (Kaikkonen et al., 2008, 2010). In our study, participants performed exhaustive exercise in the evening; therefore, the observed increases in HR and decreases in HRV during sleep were not unexpected. These responses may be related to the reduced proportion of REM sleep and the increased WASO. The reduction in REM sleep observed in EX is consistent with findings from recent meta‐analyses (Frimpong et al., 2021; Yue et al., 2022). Furthermore, Aloulou et al. (2020) reported that high‐intensity running performed at 9:00 pm increased WASO during the first 180 min of sleep and was accompanied by elevated HR throughout the night. However, in the present study, the decreased HRV parameters in EX were limited to the first 90 min of sleep. Notably, SOL and the duration of stage N3 sleep were not adversely affected during this period. In EX, a sufficient decline in CBT before bedtime may have helped to maintain SOL and the duration of stage N3 sleep, despite the alterations in HR and parasympathetic nervous activity. Nevertheless, the transient elevation in CBT during the first hour of sleep should be considered when interpreting the present findings.

Our study has several limitations. First, the sample size (*n* = 12) was relatively small, which may have limited our ability to detect subtle differences between conditions. Especially, sensor‐related recording failures reduced the number of participants available for EEG (*n* = 11) and HRV (*n* = 9) analyses. Therefore, smaller effects on some sleep and autonomic variables may not have been detected. Second, the data were obtained from male swimmers. Inclusion of female participants might have yielded different findings. Third, hormonal and inflammatory responses, which may influence sleep quality (Åkerstedt & Nilsson, 2003), were not evaluated. Moreover, sleep quality following high‐intensity exercise in water was not compared with that following high‐intensity exercise on land. Therefore, it cannot be determined whether the present findings are specific to high‐intensity exercise performed in water (swimming exercise). Finally, it is possible that participants in CON who conducted daily training before the experimental day may not have fully recovered from residual fatigue, which may have negatively affected sleep quality through elevated cortisol secretion (Kirwan et al., 1988).

## 5 CONCLUSION

Although evening swimming exercise significantly increased CBT during swimming, no significant difference in CBT at bedtime was observed between the conditions. Following swimming exercise, WASO increased significantly and was accompanied by elevated HR throughout the night and reduced parasympathetic nervous activity during the initial phase of sleep. Therefore, certain aspects of sleep architecture were impaired following high‐intensity swimming exercise. However, other sleep variables, including SE, SOL, and the duration and percentages of N3 sleep, were preserved. These findings suggest that evening high‐intensity swimming exercise induces selective rather than generalized impairments in sleep.

## AUTHOR CONTRIBUTIONS

**Hiroki Furukawa:** Conceptualization; data curation; formal analysis; investigation; methodology. **Tomotaka Natsui:** Data curation; formal analysis; investigation. **Takenobu Chugun:** Data curation; formal analysis; investigation. **Chihiro Tomiishi:** Data curation; formal analysis; investigation. **Rin Mizuno:** Data curation; formal analysis; investigation. **Yuri Maeoka:** Data curation; formal analysis; investigation. **Kazushige Goto:** Conceptualization; data curation; investigation; project administration; supervision.

## FUNDING INFORMATION

The authors have nothing to report.

## CONFLICT OF INTEREST STATEMENT

The authors declare no conflict of interest.

## ETHICS STATEMENT

The Ethics Committee for Human Experiments at Ritsumeikan University (BKC‐LSMH‐2024‐038) approved the study protocol. The study was performed in accordance with the Declaration of Helsinki.

## Data Availability

The data and analyses that support the findings of this study are not publicly available. The datasets are available from the corresponding author on reasonable request.
